# Two host microRNAs influence WSSV replication via *STAT* gene regulation

**DOI:** 10.1038/srep23643

**Published:** 2016-03-31

**Authors:** Ying Huang, Wen Wang, Qian Ren

**Affiliations:** 1 Jiangsu Key Laboratory for Biodiversity & Biotechnology and Jiangsu Key Laboratory for Aquatic Crustacean Diseases, College of Life Sciences, Nanjing Normal University, Nanjing 210046, China

## Abstract

MicroRNAs (miRNAs) have important roles in post-transcriptional regulation of gene expression. During viral infection, viruses utilize hosts to enhance their replication by altering cellular miRNAs. The Janus kinase (JAK)/signal transducer and activator of transcription (STAT) pathway plays crucial roles in the antiviral responses. In this study, two miRNAs (miR-9041 and miR-9850) from *Macrobrachium rosenbergii* were found to promote white spot syndrome virus (WSSV) replication. The up-regulation of miR-9041 or miR-9850 suppresses *STAT* expression in the gills of *M. rosenbergii*, which subsequently down-regulates the expression of its downstream dynamin (*Dnm*) genes: *Dnm1*, *Dnm2*, and *Dnm3*. Knockdown of miR-9041 and miR-9850 restricts WSSV replication by up-regulating *STAT* and *Dnm* gene expression. The silencing of *STAT*, *Dnm1*, *Dnm2*, or *Dnm3* led to an increase of the number of WSSV copies in shrimp. The injection of recombinant Dnm1, Dnm2, or Dnm3 proteins could inhibit WSSV replication *in vivo*. Overall, our research indicates the roles of host miRNAs in the enhancement of WSSV replication by regulating the host JAK/STAT pathway.

The giant fresh water prawn, *Macrobrachium rosenbergii*, has become an important cultured species in China and other southern East Asian countries^1^,^2^. With the development of intensive culture and environmental deterioration, serious infectious diseases caused by viruses have been reported in *M. rosenbergii*^3^. Among these viruses, the white spot syndrome virus (WSSV) has emerged as one of the most prevalent and widespread viruses^4^,^5^. WSSV is a bacilliform, non-occluded enveloped virus, which belongs to the genus *Whispovirus* of the Nimaviridae family^6^,^7^. WSSV is a large double-stranded DNA virus with a virion that consists of a nucleocapsid, tegument, and envelope and includes at least 39 structural proteins^8^,^9^. WSSV remains a major threat to shrimp aquaculture^10^.

The Toll, immune deficiency (IMD), and Janus kinase (JAK)/signal transducer and activator of transcription (STAT) pathways are reported to regulate the humoral immunity of shrimp^11^. Limited information is available regarding the role of the JAK/STAT pathway during WSSV infection^11^. The JAK/STAT pathway was first identified as a cytokine-induced signaling pathway in mammals; to date, the pathway is known to be involved in immune response and inflammation, particularly in the interferon-mediated antiviral response in mammals^12^,^13^. Infectious viruses induce the production of interferons, interleukins, and growth factors, among others^14^. These compounds are recognized by cytokine receptors, which activate the JAK/STAT pathway and leads to the transcription of interferon-inducible genes with antiviral functions^15^. In humans, the four JAKs (JAK1, JAK2, JAK3, and TYK2) and seven STATs (STAT1, STAT2, STAT3, STAT4, STAT5A, STAT5B, and STAT6) mediate responses to a number of cytokines and activate different downstream genes^16^. In *Culex* mosquitoes, the activation of the JAK/STAT pathway can induce the expression of *Vago*, which is regarded as an antiviral factor, to restrict the West Nile virus infection^17^. In *Drosophila*, this pathway activates at least two gene families: thioester-containing protein (TEP) and TOT, which are involved in innate immunity^18^. In *Penaeus monodon*, STAT directly transactivates the expression of the WSSV immediate-early gene *ie1* and contributes to its high promoter activity^19^.

In humans, the myxovirus resistance gene *MxA* belongs to the superfamily of dynamin-like large GTPases. *MxA* is reported to be JAK/STAT-dependent and can inhibit the replication of orthomyxoviruses^20^. Dynamin (Dnm) is a large multi-domain GTPase essential for the membrane fission leading to clathrin-mediated endocytosis^21^. Dnm has three isoforms in mammalian cells: Dnm1, Dnm2, and Dnm3, which are encoded by distinct genes that are differentially expressed in various tissues^22^. Dnm is a key mediator of cell-autonomous innate immunity against a broad range of viruses^23^. In addition to its involvement in virus endocytosis, Dnm has also been proposed to participate in membrane fusion between viruses and endosomes after endocytosis^24^.

MicroRNAs (miRNAs) have roles in the post-transcriptional regulation of gene expression^25^ and various biological processes, such as proliferation, cell differentiation, apoptosis, tumorigenesis, and immune defense^25^,^26^. The production of mature miRNAs requires the participation of several molecules^27^,^28^. Typically, targeted mRNA leads to translation repression and/or mRNA degradation^29^. More miRNAs have been shown to participate in innate and adaptive immune response during virus infection by regulating the viral or host gene expression^30^. In humans, the Epstein–Barr virus (EBV)-encoded viral miR-BART22 modulates the viral gene product expression of the EBV latent membrane protein 2A (LMP2A), which may facilitate nasopharyngeal carcinoma carcinogenesis by evading the host immune response^31^. A human herpes virus miR-K12-11 attenuates IFN signaling and contributes to maintenance of viral latency by targeting I-κ-B kinase epsilon (IKKɛ)^32^. In *Marsupenaeus japonicus* shrimp, the viral miRNAs WSSV-miR-66 and WSSV-miR-68 could target WSSV genes and further promote WSSV infection^33^. However, the host miRNA-mediated regulations of the STAT gene and its downstream genes have not been well studied in the giant freshwater prawn to date.

In this study, we demonstrated that miR-9041 and miR-9850 played positive roles in WSSV replication. The up-regulation of miR-9041 or miR-9850 suppresses *STAT* expression in the gills of *M. rosenbergii*, which subsequently down-regulates the expression of its downstream *Dnm* genes. The RNA interference (RNAi) of *Dnm* genes or the overexpression of Dnms by injection of recombinant proteins could enhance or inhibit virus replication, respectively. Our research describes the roles of host miRNAs in enhancing WSSV replication by regulating the host JAK/STAT pathway.

## Results

### Effects of miR-9041 and miR-9850 on virus infection in shrimp

**Figure 1 f1:**
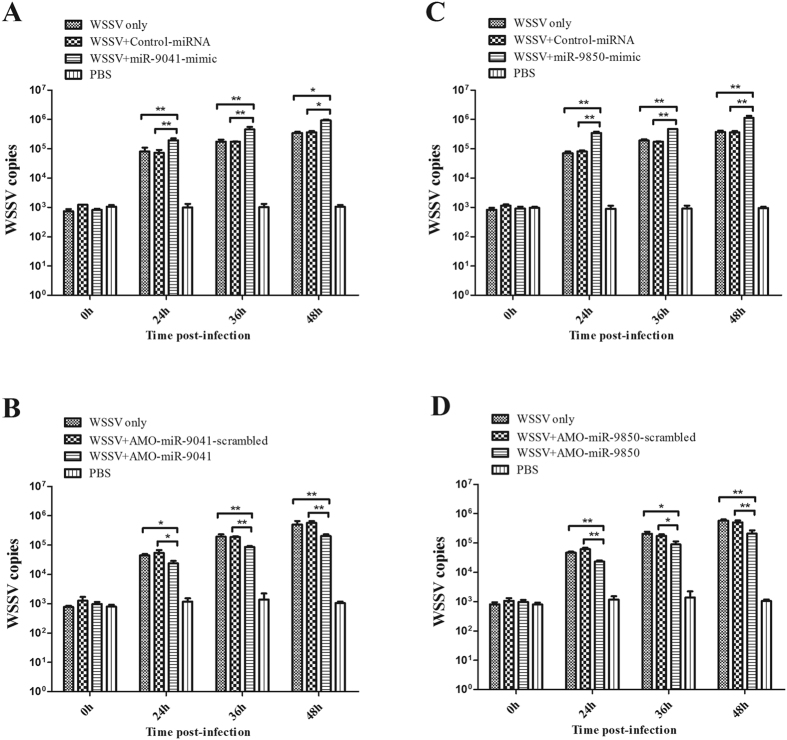
Roles of host miRNAs on virus infection in shrimp *in vivo*. The effect of miR-9041 (**A**) or miR-9850 (**C**) overexpression on virus infection in the gills of shrimp. To overexpress the host miRNA, miR-9041-mimic or miR-9850-mimic and WSSV were co-injected into shrimp. At different time post-infection (0, 24, 36, and 48 h), the WSSV copies in shrimp were examined. U6 was used as a control. The influence of miR-9041 (**B**) or miR-9850 (**D**) silencing on the virus infection *in vivo*. AMO-miR-9041 or AMO-miR-9850 and WSSV were co-injected into shrimp. At various time post-infection (0, 24, 36, and 48 h), the WSSV copies in shrimp were evaluated. The numbers showed the time points after WSSV infection in shrimp. Asterisks indicated significant differences (*p < 0.05; **p < 0.01) between treatments.

These 2 microRNAs (miR-9041 and miR-9850) was inducibly expressed in the WSSV challenged group in relative to the normal group (without WSSV challenge) based on the small RNA high-throughput sequencing data. So, we selected these 2 microRNAs for functional study. To further elucidate the roles of the host miR-9041 and miR-9850 in virus infection, both miRNAs were overexpressed in shrimp. When miR-9041 or miR-9850 was overexpressed in shrimp, the number of WSSV copies was examined. As shown in Fig. 1A, the overexpression of miR-9041 significantly increased the number of WSSV copies from 24 h to 48 h post-infection compared with the controls (miR-9041-scrambled and WSSV only). The overexpression of miR-9850 yielded similar results (Fig. 1C). By contrast, when miR-9041 or miR-9850 expression was knocked down by sequence-specific AMO-miR-9041 and AMO-miR-9850 *in vivo*, respectively, the WSSV copies were significantly decreased as compared with the controls (AMO-miR-9041-scrambled, AMO-miR-9850-scrambled, and WSSV only; Fig. 1B[Fig f1]D). These findings indicated that the host miRNAs play a positive role in virus replication.

### Interaction between the host miRNAs and the host *STAT* gene

**Figure 2 f2:**
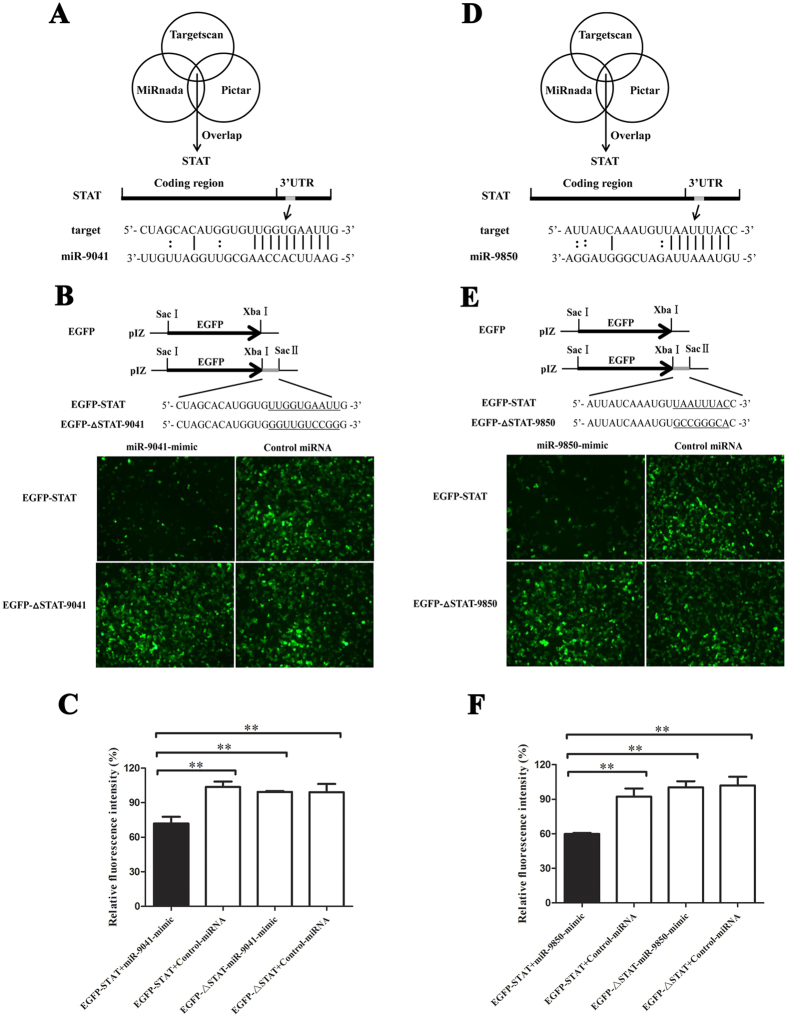
The interaction between miR-9041 or miR-9850 and host *STAT* gene *in vitro*. The predicted target gene of miR-9041 (**A**) or miR-9850 (**D**). The 3′UTR of the *STAT* gene could be targeted by miR-9041 and miR-9850. The direct interaction between miR-9041 (**B**) or miR-9850 (**E**) and host gene. The synthesized miR-9041-mimic or miR-9850-mimic and the plasmid consisting of EGFP and shrimp *STAT* 3′UTR were co-transfected into insect High Five cells. The mutant of *STAT* 3′UTR (EGFP-∆STAT-9041 or EGFP-∆STAT-9850) was included in the co-transfection as a control. At 48 h after cotransfection, the fluorescence of cells was examined. The seed sequences targeted by miR-9041 and miR-9850 were underlined. Effects of miR-9041 (**C**) or miR-9850 (**F**) on the *STAT* gene expression. The relative fluorescence intensity of cells was determined. Statistically significant differences between treatments were indicated by asterisks (**p < 0.01).

Increasing evidence has indicated that host miRNAs has important roles in host–virus interactions. To reveal the pathways mediated by host miRNAs, the target genes of miR-9041 and miR-9850 were analyzed. The predictions by the TargetScan, miRanda, and Pictar algorithms showed that miR-9041 and miR-9850 could target the host *STAT* gene (Fig. 2A[Fig f2]D), which is a gene involved in the antiviral immunity of shrimp^19^. Therefore, these host miRNAs may have significant effects on WSSV infection in shrimp.

To evaluate the interaction between the host miR-9041 or miR-9850 and the host *STAT* gene, we constructed the EGFP-STAT plasmid, which contained EGFP and the 3′-UTR of *STAT* in *M. rosenbergii* shrimp. The synthesized miR-9041 or miR-9850 mimic and the plasmid EGFP-STAT were co-transfected into insect High Five cells (Fig. 2B[Fig f2]E). The results showed that the fluorescence intensity in the co-transfected cells was significantly reduced compared with the intensity in the EGFP-STAT-transfected cells (Fig. 2C[Fig f2]F), thereby indicating that the synthesized miR-9041 or miR-9850 mimic repressed the expression of the STAT gene by targeting its 3′-UTR. However, the control miRNA mimic had a negligible effect on the expression of EGFP-STAT.

**Figure 3 f3:**
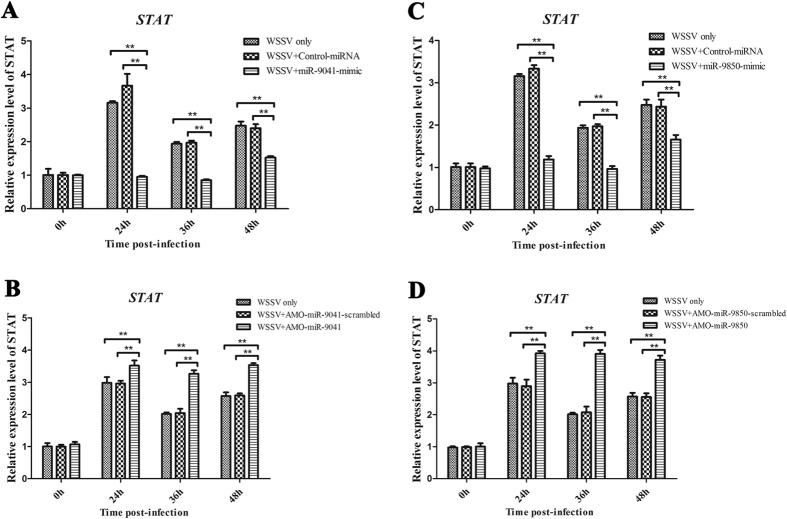
The interaction between miR-9041 or miR-9850 and host *STAT* gene *in vivo*. The influence of miR-9041 (**A**) or miR-9850 (**C**) overexpression on the expression of shrimp *STAT* gene *in vivo*. miR-9041-mimic or miR-9850-mimic and WSSV were co-injected into shrimps. As a control, WSSV only was included in the injections. At different time post-infection (0, 24, 36, and 48 h), the expression of *STAT* was examined with quantitative real-time PCR. The effect of miR-9041 (**B**) or miR-9850 (**D**) silencing on the *STAT* expression *in vivo*. AMO-miR-9041 or AMO-miR-9850 was injected into WSSV-infected shrimp to knock down the miR-9041 or miR-9850 expression. WSSV only was used as a control. At different time post-infection (0, 24, 36, and 48 h), the expression of *STAT* was detected with quantitative real-time PCR. Statistically significant differences between treatments were indicated by asterisks (**p < 0.01).

To further explore the interaction between miR-9041 or miR-9850 and *STAT in vivo*, the expression of miR-9041 and miR-9850 was overexpressed or silenced in shrimp, followed by the detection of *STAT* mRNA levels. The results indicated that miR-9041 or miR-9850 overexpression significantly decreased the STAT expression compared with the positive control (WSSV only), whereas the control miRNAs had no effect on the expression of *STAT*, thereby showing that miR-9041 and miR-9850 inhibited *STAT* expression in shrimp (Fig. 3A[Fig f3]C). When the expression of miR-9041 or miR-9850 was knocked down by AMO-miR-9041 or AMO-miR-9850, the expression of *STAT* was significantly up-regulated (Fig. 3B[Fig f3]D). These findings showed that miR-9041 and miR-9850 could interact with the host *STAT* gene *in vivo*.

### Role of host STAT in virus infection

**Figure 4:**
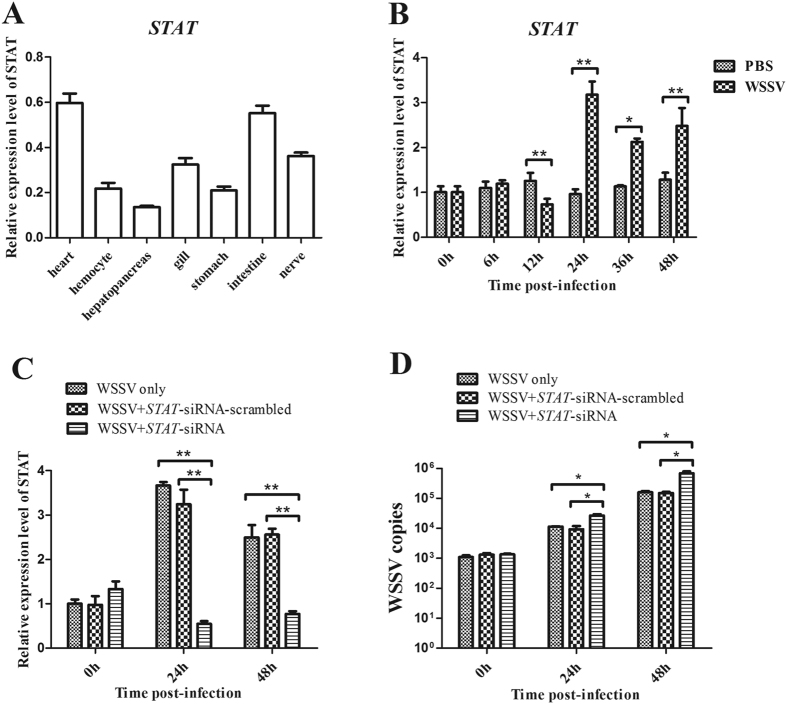
The role of host STAT in virus infection. (**A**) The distribution of *STAT* in various tissues of shrimp. The shrimp *STAT* mRNA levels of heart, hemocyte, hepatopancreas, gill, stomach, intestine and nerve were examined using quantitative real-time PCR. Shrimp GAPDH was used as a control to calibrate the cDNA template for all the samples. (**B**) The expression profile of *STAT* in shrimp in response to virus infection. Shrimp were infected with WSSV. At different time post-infection (0, 6, 12, 24, 36, and 48 h), the *STAT* expression in gills was detected with quantitative real-time PCR. (**C**) The silencing of the *STAT* expression in shrimp. The sequence-specific STAT-siRNA was injected into shrimp to knock down the expression of *STAT*. As a control, STAT-siRNA-scrambled was included in the injections. WSSV alone was used as a positive control. (**D**) The influence of STAT silencing on virus infection. The WSSV copies in gills of STAT-siRNA-treated shrimp were quantified using quantitative real-time PCR. In all panels, statistically significant differences between treatments were indicated by asterisks (**p < 0.01).

The abovementioned data showed that the host *STAT* gene is the target of miR-9041 and miR-9850. Therefore, the role of *STAT* in virus infection was explored in shrimp. Results of qRT-PCR indicated that the *STAT* mRNA was detected in all the examined tissues and mainly expressed in the heart and intestine tissues of shrimp (Fig. 4A). In response to WSSV infection, the expression level of *STAT* was up-regulated from 24 h to 48 h, which was significantly higher than that of the untreated controls (Fig. 4B). The results suggested that the *STAT* gene may play an important role in virus infection.

To evaluate the influence of STAT in WSSV infection, the *STAT* expression was silenced by gene-specific siRNA (STAT-siRNA) in shrimp *in vivo*, followed by the detection of virus copies. The expression of the *STAT* gene was significantly knocked down at 24 and 48 h post-infection compared with the WSSV-only control group; by contrast, STAT-siRNA-scrambled had no effect on the *STAT* expression (Fig. 4C), thereby showing that the siRNA was highly specific. When the expression of the *STAT* gene was knocked down, the number of WSSV copies in shrimp gills was significantly increased compared with the control (WSSV only; Fig. 4D). The data indicated that the host *STAT* gene plays a negative role in WSSV infection *in vivo*.

### *Dnm1*, *Dnm2*, and *Dnm3* expression is regulated by *STAT*

**Figure 5:**
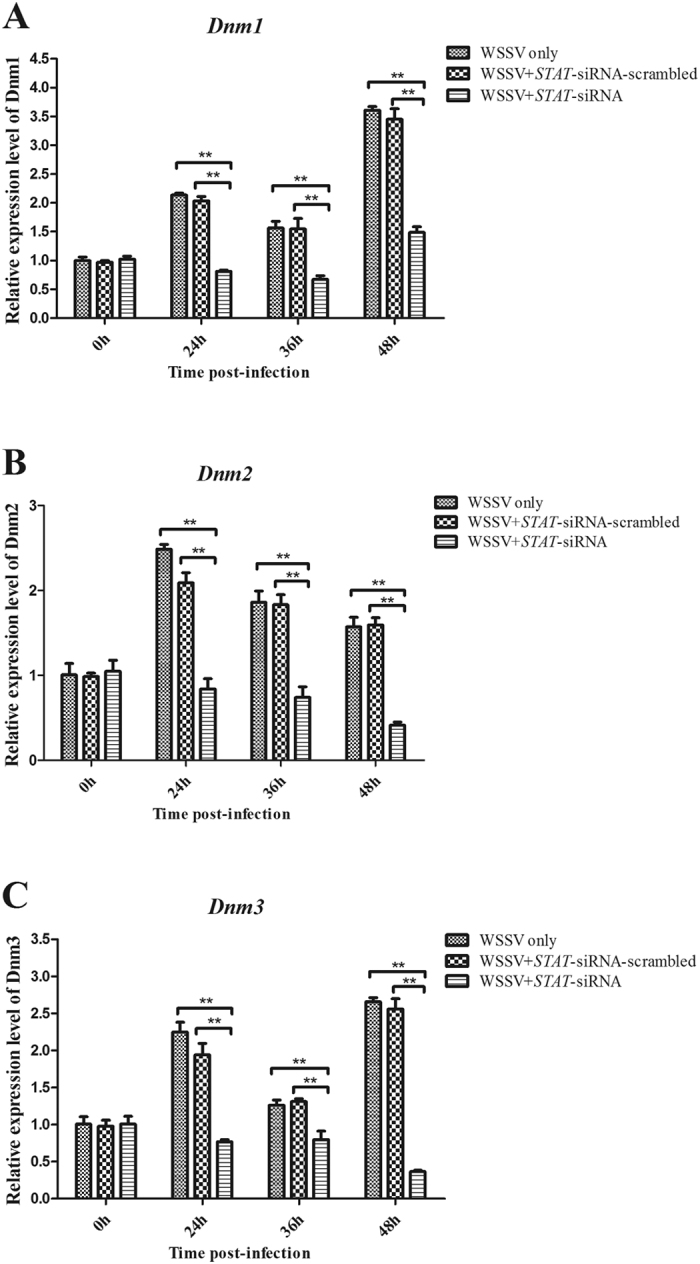
Interaction between *STAT* gene and its downstream Dnm genes. The WSSV-infected shrimp were injected with STAT-siRNA to silence the expression of *STAT* gene, followed by the quantification of *Dnm1* (**A**), *Dnm2* (**B**) or *Dnm3* (**C**) mRNA using quantitative real-time PCR. The results are shown as mean and standard deviation from three independent experiments (**p < 0.01).

The human *Mx* homolog *MxA* belongs to the class of interferon (IFN)-stimulated genes (ISGs); this gene is JAK/STAT-dependent and can inhibit the replication of orthomyxoviruses and other RNA viruses^21^. Haller and Kochs reported that Mx proteins belong to the superfamily of Dnm-like large GTPases^21^. Consequently, we predicted that Dnms are important effectors of the JAK-STAT signaling pathway in *M. rosenbergii*. To reveal the relationship between *STAT* and the *Dnm* genes (*Dnm1*, *Dnm2*, and *Dnm3*), the expression of *STAT* was knocked down by *STAT*-siRNA in shrimp. After the expression of the *STAT* gene was silenced, the *Dnm1*, *Dnm2*, and *Dnm3* expression levels were detected by qRT-PCR. The results are shown in Fig. 5, wherein the three *Dnms* were induced by 1.4-fold to 3.5-fold after the WSSV challenge. However, the expression levels of these *Dnms* were significantly decreased in the *STAT*-siRNA experimental group compared with the controls (Fig. 5A–C). Therefore, *Dnm1*, *Dnm2*, and *Dnm3* are likely the downstream genes of *STAT*.

### *Dnm1*, *Dnm2*, and *Dnm3* mediated by miR-9041 or miR-9850

**Figure 6:**
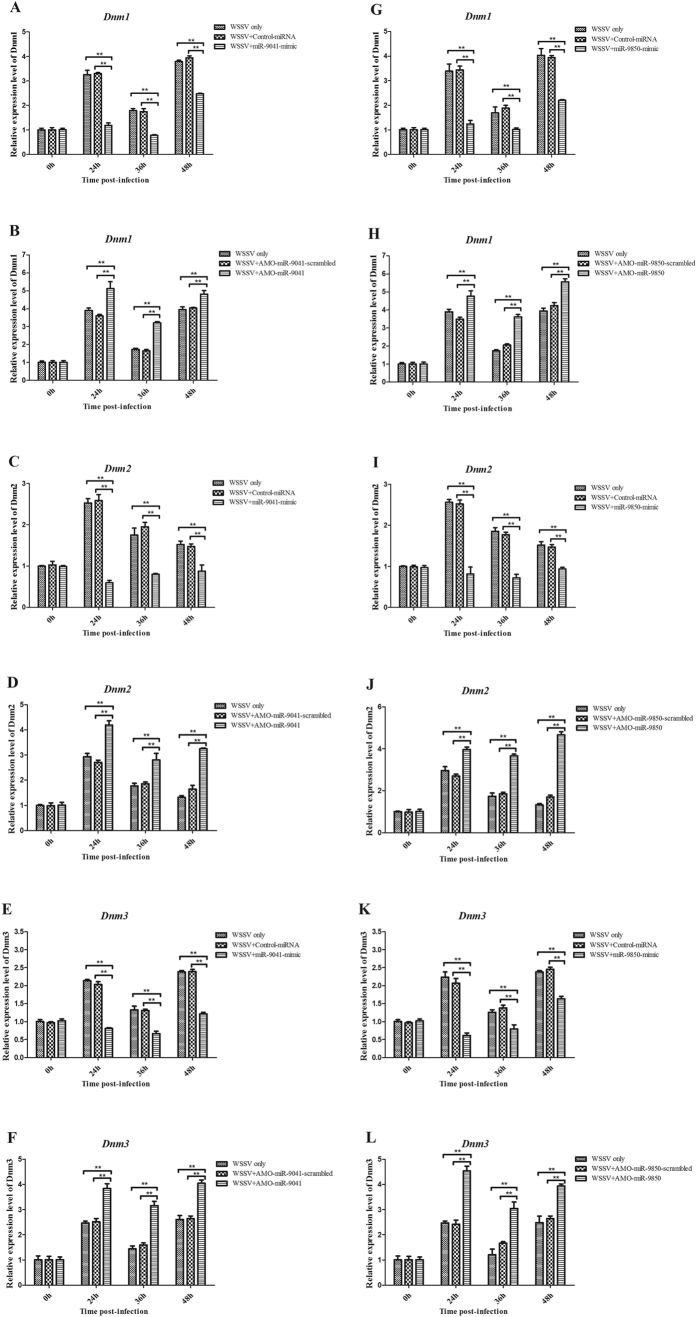
The pathway mediated by miR-9041 or miR-9850 in virus infection. Effects of miR-9041 overexpression on the expressions of *Dnm1* (**A**), *Dnm2* (**C**) or *Dnm3* (**E**) *in vivo*. Effects of miR-9850 overexpression on the expressions of *Dnm1* (**G**), *Dnm2* (**I**) or *Dnm3* (**K**). miR-9041-mimic or miR-9850-mimic and WSSV were co-injected into shrimp. As a control, WSSV only was included in the injections. At different time after injection (0, 24, 36, and 48 h), the mRNA levels of *Dnm1*, *Dnm2* and *Dnm3* were quantified by quantitative real-time PCR. Influence of miR-9041 silencing on the *Dnm1* (**B**), *Dnm2* (**D**) or *Dnm3* (**F**) expressions. Influence of miR-9850 silencing on the *Dnm1* (**H**), *Dnm2* (**J**) or *Dnm3* (**L**) expressions. AMO-miR-9041 or AMO-miR-9850 was injected into WSSV-infected shrimp to knock down the miR-9041 or miRv-9850 expression. WSSV only was used as a control. At different time post-infection (0, 24, 36, and 48 h), the expressions of *Dnm1*, *Dnm2* and *Dnm3* were detected with quantitative real-time PCR. Statistically significant differences between treatments were indicated by asterisks (**p < 0.01).

The abovementioned data implied that miR-9041, miR-9850, STAT, Dnm1, Dnm2, and Dnm3 are involved in the virus infection of shrimp *in vivo*. Therefore, the pathway mediated by the host miR-9041 and miR-9850 was further explored. The results indicated that the expression levels of *Dnm1*, *Dnm2*, and *Dnm3* are significantly down-regulated when miR-9041 or miR-9850 is overexpressed in shrimp (Fig. 6A,C,E,G,I,K). When the expression of miR-9041 or miR-9850 is knocked down by AMO-miR-9041 or AMO-miR-9850, the expression levels of *Dnm1*, *Dnm2*, and *Dnm3* are up-regulated (Fig. 6B,D,F,H,J,L), thereby suggesting that the host miRNAs, Dnm1, Dnm2, and Dnm3 shared the same pathway.

### Effects of *Dnm1*, *Dnm2*, and *Dnm3* in virus infection

**Figure 7:**
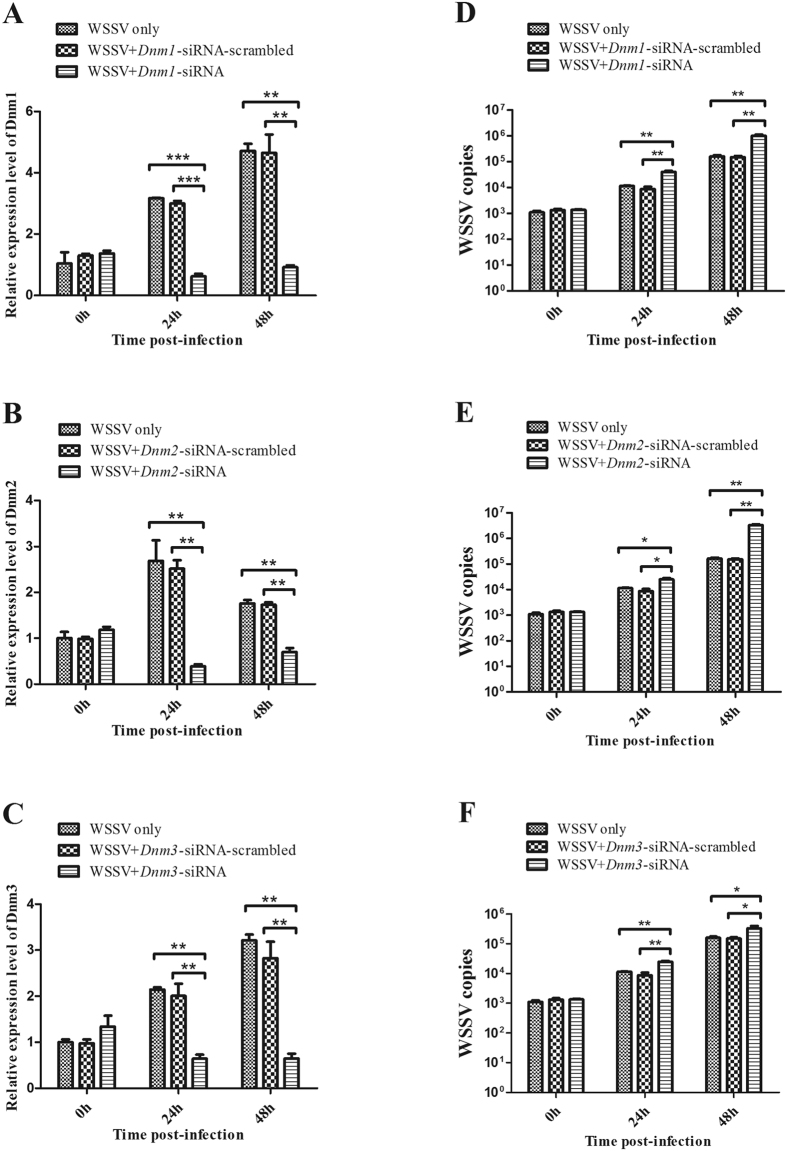
The effects of host Dnm1 to Dnm3 in mRNA level on virus infection. The gene expression silencing of *Dnm1* (**A**), *Dnm2* (**B**) or *Dnm3* (**C**) in shrimp. The sequence-specific *Dnm1*-siRNA, *Dnm2*-siRNA or *Dnm2*-siRNA was injected into shrimp. Then the gene expression was examined. As a negative control, siRNA-scrambled was included in the injections. WSSV alone was used as a positive control. The detection of WSSV copies after *Dnm1* (**D**), *Dnm2* (**E**) or *Dnm3* (**F**) knocked down in shrimp. The gills of siRNA-treated shrimp were subjected to the quantitative real-time PCR analysis to examine the WSSV copies. WSSV alone was used as a positive control. In all panels, asterisks indicated significant differences between treatments (**p < 0.01).

As one of the key effectors of the JAK/STAT pathway, *Dnm* genes are downstream of *STAT*. To evaluate the roles of *Dnm1*, *Dnm2*, and *Dnm3* in viral infection, these genes were characterized in shrimp. As shown in Figs. S1A, S1C, and S1E, the mRNAs of *Dnm1*, *Dnm2*, and *Dnm3* were detected in all the examined tissues, including the hemocyte, heart, hepatopancreas, gills, stomach, intestine, and nerve tissues. In response to WSSV infection, the expression levels of *Dnm1*, *Dnm2*, and *Dnm3* were significantly up-regulated from 24 h to 48 h (Figs. S1B, S1D, and S1F). Furthermore, when the expression of *Dnm1*, *Dnm2*, and *Dnm3* was knocked down by sequence-specific siRNAs (Fig. 7A–C), the number of WSSV copies significantly increased as compared with the controls (WSSV only). By contrast, the *Dnm1*-siRNA-scrambled, *Dnm2*-siRNA-scrambled, and *Dnm3*-siRNA-scrambled treatments had no effect on the virus infection (Fig. 7D–F). The data demonstrated that *Dnm1*, *Dnm2*, and *Dnm3* had significant effects on virus infection in shrimp.

**Figure 8:**
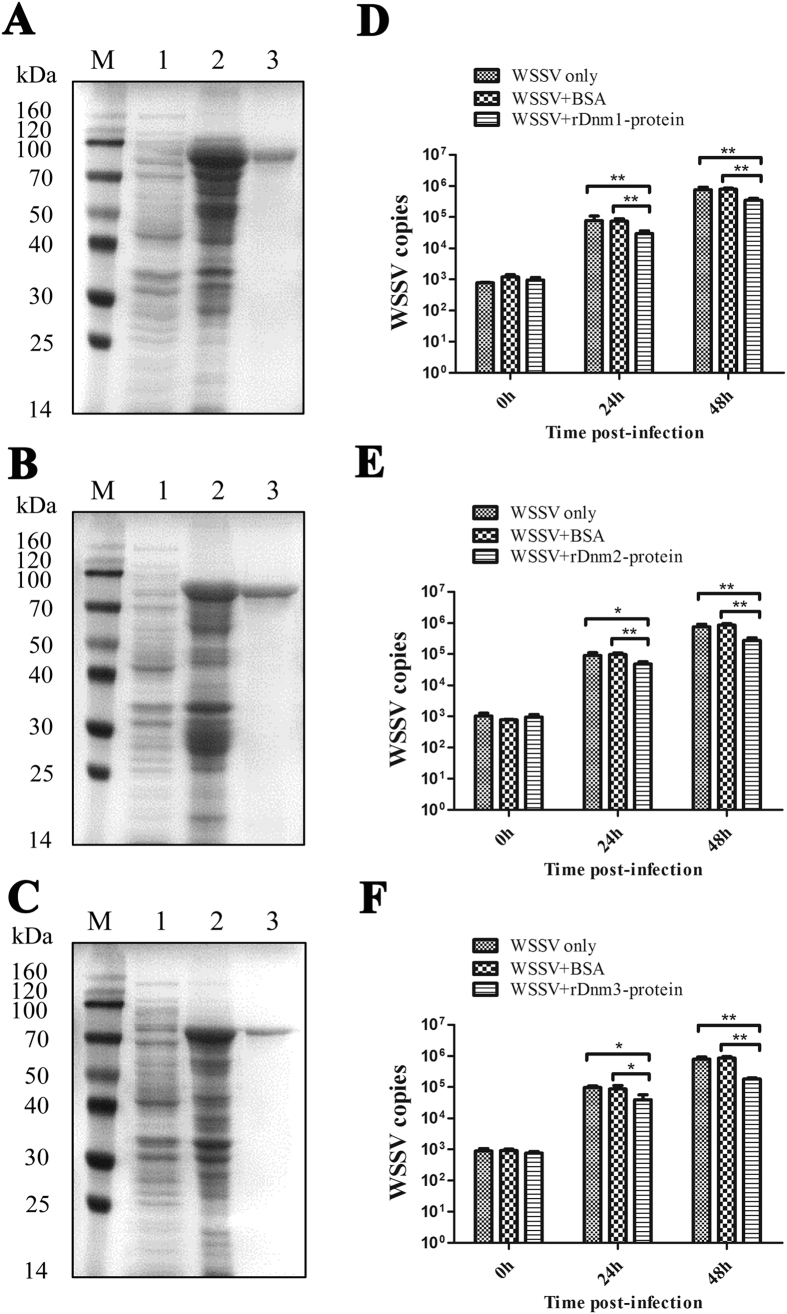
The effects of host Dnm1 to Dnm3 in protein level on virus infection. SDS–PAGE analysis of rDnm1 (**A**), rDnm2 (**B**) and rDnm3 (**C**). Lane M: protein molecular weight standards (kDa); lane 1: negative control (without induction); lane 2: IPTG induced rDnms; lane 3: purified rDnms. Recombined Dnm1 (**D**), rDnm2 (**E**) and rDnm3 (**F**) proteins affect the WSSV copies in shrimp. Purified rDnm1, rDnm2 or rDnm3 was individually incubated with WSSV at 28 °C for 30 min with rotation. BSA as control was incubated with WSSV. Four groups of mixtures were injected into the shrimps. The gills of shrimps were collected at different post injection times (0, 24, and 48 h) to detect the WSSV copies by quantitative real-time PCR. Asterisks indicated significant differences (*p < 0.05; **p < 0.01) between treatments.

Recombinant plasmids (pET30a-Dnm1, pET30a-Dnm2, and pET30a-Dnm3) were respectively transformed into *E. coli* BL21 (DE3). After IPTG induction for 4 h, the whole cell lysates were analyzed by SDS–PAGE. In Fig. 8A, a distinct band with a molecular weight (MW) of approximately 100 kDa was detected, which was roughly consistent with the predicted MW of Dnm1 (theoretical MW, 96.4 kDa) and the ~5 kDa His tag at the N-terminal. The recombinant Dnm2 contained an additional N-terminal His Tag (5 kDa); thus, its size was relatively larger (~90 kDa) than the theoretical MW (84.8 kDa; Fig. 8B). Furthermore, a distinct band with an 80 kDa molecular mass was revealed, which agreed with the predicted molecular mass of the recombinant Dnm3 protein (77.6 kDa) with a His tag (Fig. 8C).

Given that *Dnm1*, *Dnm2*, and *Dnm3* could be involved in virus infection at the mRNA level, we further hypothesized whether or not recombined Dnm1, Dnm2, and Dnm3 proteins affect the WSSV copies in shrimp. To test this hypothesis, the inhibition of WSSV replication was performed. The number of WSSV copies was replicated at a slower rate in the gills of the rDnm1, rDnm2, or rDnm3 injection groups compared with the BSA control and WSSV only groups at all post-injection times (24 and 48 h; Fig. 8D–F). These findings indicated that rDnm1, rDnm2, and rDnm3 had important functions in the inhibition of WSSV replication.

## Discussion

MicroRNAs play an important role in the regulation of gene expression^25^. First discovered in the nematode *Caenorhabditis elegans*, miRNAs have been identified in all multicellular eukaryotes and some viruses^34^. Recent studies demonstrate that miRNAs can also strongly affect the replication of pathogenic viruses. Viruses are known to encode their own miRNAs and/or trigger changes in cellular miRNA expression to target mRNAs of virus and/or host genes, which further results in either mRNA degradation or translational repression^33^. The viral miRNAs WSSV-miR-66 and WSSV-miR-68 can promote WSSV infection by targeting the WSSV genes (the WSSV *wsv094* and *wsv177* genes are the targets of WSSV-miR-66; the *wsv248* and *wsv309* genes are the targets of WSSV-miR-68)^33^. WSSV-miR-N24 is employed by WSSV to regulate the expression of shrimp *caspase 8* and facilitate viral replication by inhibiting the host antiviral apoptotic activity^35^. Cellular miRNAs can influence hepatitis B virus (HBV) replication directly by binding to HBV transcripts and indirectly by targeting cellular factors relevant to the HBV life cycle^36^. In human hepatocytes, the host miR-373 is up-regulated by the hepatitis C virus during its infection and negatively regulated by the type I IFN signaling pathway via suppression of JAK1 and IFN regulating factor 9 (IRF9)^37^. The host miR-146 targets the interleukin 1 receptor-associated kinase (IRAK1) and TNF receptor-associated factor 6 (TRAF6) as a negative regulator of constitutive nuclear factor-κB (NF-κB) activity in breast cancer^38^. However, the regulation of transcription factor expression by the simultaneous mediation of two different host miRNAs has not been explored to date. Our study highlights a novel aspect of two host miRNAs, which target the mRNA of host *STAT* gene and suppress the corresponding JAK/STAT-Dnm1/Dnm2/Dnm3 signaling pathways in the virus–host interactions *in vivo*.

To date, more than 10,000 miRNAs have been annotated in 96 species^39^, including over 2500 human miRNAs (miRBase ver. 21; http://www.mirbase.org/, released in June 2014). Each miRNA can regulate hundreds of different mRNAs, whereas a single mRNA can be conversely targeted by several miRNAs^40^. Cellular miRNAs play crucial roles in several biological processes, such as the innate and adaptive immune response; the deregulation of miRNA expression and function is also involved in various diseases^41^. Viruses can alter cellular miRNA expression; cellular miRNAs can positively or negatively influence virus replication during viral infection by regulating the expression of viral genes and/or cellular factors relevant to the course of virus-induced disease^42^. Viruses are equipped with complex machinery to exploit and manipulate the host pathways to establish an environment favorable for their persistence^43^.

In this study, shrimp miR-9041 and miR-9850 can regulate the expression of the host gene *STAT*, which is one of key components of the JAK/STAT pathway. A WSSV-encoded miRNA (WSSV-miR-22) could also promote WSSV infection in *Marsupenaeus japonicas* shrimp by targeting the host *STAT* gene^44^. The JAK-STAT signaling pathway plays a critical role in the initiation of antiviral response. An IFN-like antiviral cytokine known as *Vago* can activate the JAK/STAT pathway to control viral loads in West Nile virus-infected *Culex quinquefasciatus* cells^17^. The up-regulation of miR-9041 or miR-9850 suppresses the expression of STAT and its downstream molecules to promote WSSV replication. However, the downstream genes and their precise mechanisms are not yet fully understood.

In our previous study, thioester-containing protein TEP1 and TEP2 were the effectors of JAK-STAT signaling pathway^44^. Whereas in this study, *Dnm* genes were found to be downstream of STAT. Dnms are involved in antiviral immune defense. The Mx dynamin-like GTPases are key antiviral effector proteins of the types I and III IFN systems^23^. Human Mx2 or MxB are ISGs that contribute to the inhibition of the human immunodeficiency virus (HIV-1) replication by interferons^45^. A Dnm-dependent protein-trafficking pathway can mediate Ebola virus glycoprotein toxicity^46^. Dnm is required for recombinant adeno-associated virus type 2 (rAAV-2) infections, whereas the overexpression of mutant DnmI significantly inhibited AAV-2 internalization and gene delivery^47^. Therefore, Dnm is a key mediator involved in a broad range of viral infections.

In conclusion, we demonstrated that the host miR-9041 and miR-9850 play positive roles in WSSV replication by targeting the host JAK/STAT signal pathway. *Dnm* genes are regulated by STAT and are involved in antiviral immune response. Therefore, WSSV infection could induce host miRNAs, which inhibit the JAK/STAT pathway and finally enhance virus replication.

## Materials and Methods

### Shrimp culture and WSSV challenge

The *M. rosenbergii* shrimp (approximately 15 g each) were purchased from an aquaculture market in Nanjing, Jiangsu Province, China, and acclimatized under laboratory conditions in the freshwater tanks at room temperature (25 °C) for a week before processing. To ensure that the shrimp were WSSV-free before experiments, PCR was conducted with WSSV-specific primers (5′-TATTGTCTCTCCTGACGTAC-3′and 5′-CACATTCTTCACGAGTCTAC-3′). Various tissues (hemocyte, heart, hepatopancreas, gills, stomach, intestine, and nerve; from 5 virus-free shrimp as parallel samples) were collected to determine the tissue distribution of *STAT*, *Dnm1*, *Dnm2*, and *Dnm3* transcripts. Hemolymph was collected from untreated shrimp by mixing half the volume of anticoagulant buffer (0.14 M NaCl, 0.1 M glucose, 30 mM trisodium citrate, 26 mM citric acid, and 10 mM EDTA; pH 4.6)^48^. Samples were immediately centrifuged at 800 *×* *g* at 4 °C for 15 min to harvest the hemocytes. A total of 20 healthy shrimp were infected with 100 μL of WSSV virus solution at 10^5^ copies/mL by intramuscular injection into the lateral area of the fourth abdominal segment. At different times post-infection (0, 24, 36, and 48 h), gills of 5 shrimp were randomly collected for each treatment and stored for future use.

### Overexpression or silencing of miR-9041 and miR-9850 in shrimp

Based on their sequences, miR-9041 (5′-GAAUUCACCAAGCGUUGGAUUGUU-3′) and miR-9850 (5′-UGUAAAUUAGAUCGGGUAGGA-3′) were synthesized with the *in vitro* transcription T7 kit for siRNA synthesis (Takara, Japan). The sequences of miR-9041 and miR-9850 were scrambled to generate the miR-9041-scrambled (5′-UCAUCAGCAUCGAUAUGUAGUUGG-3′) and miR-9850-scrambled (5′-AUAGGAUAUGGGGUAUCAGUA-3′) controls. The synthesized miRNA was dissolved in miRNA solution (50 mM Tris-HCl, 100 mM NaCl, pH 7.5) and quantified by obtaining the absorbance at 260 nm with a spectrophotometer. To overexpress the host miRNAs, the miR-9041 or miR-9850 (15 μg) and WSSV (10^5^ copies/mL) were co-injected into virus-free shrimp at a volume of 100 μL/shrimp. At 16 h after co-injection, the miRNAs (15 μg; 100 μL/shrimp) were injected into the same shrimp. miR-9041-scrambled, miR-9850-scrambled, WSSV alone (10^5^ copies/mL), and phosphate buffer saline PBS (0.14 M NaCl, 3 mM KCl, 8 mM Na_2_HPO_4_, 1.5 mM KH_2_PO_4_, pH 7.4) were injected as controls.

To knock down miR-9041 or miR-9850 expression, an anti-miRNA oligonucleotide (AMO) was injected into WSSV-infected shrimp. AMO-miR-9041 (5′-AACAATCCAACGCTTGGTGAATTC-3′) was synthesized (Sangon Biotech, Shanghai, China) with a phosphorothioate backbone and a 2′-O-methyl modification at the 6th, 18th, and 22nd nucleotides. AMO-miR-9850 (5′-TCCTACCCGATCTAATTTACA-3′) was synthesized with a phosphorothioate backbone and a 2′-O-methyl modification at the 6th, 16th, and 18th nucleotides. AMO-miR-9041 or AMO-miR-9850 (10 nM) and WSSV (10^5^ copies/mL) were co-injected into virus-free shrimp at 100 μL/shrimp. At 16 h after the co-injection, AMOs (10 nM; 100 μL/shrimp) were injected into the same shrimp. AMO-miR-9041-scrambled (5′-CATGGTAAATCGACACTTCGCTAA-3′), AMO-miR-9850-scrambled (5′-TACGATCTACTCCTACAATCT-3′), WSSV alone (10^5^ copies/mL), or PBS were injected into shrimp as controls.

For each treatment, 20 shrimp were used. At different times post-infection (0, 24, 36, and 48 h), 5 shrimp were randomly collected for each treatment and subjected to the subsequent analyses. All the experiments were biologically replicated three times.

### Detection of WSSV copies by quantitative real-time PCR (qRT-PCR)

qRT-PCR was performed to examine the WSSV copies in gills of WSSV-infected shrimp. The viral DNA was extracted from shrimp gills with the EasyPure Marine Animal Genomic DNA Kit (TransGen Biotech, Beijing, China). The WSSV copies were detected by qRT-PCR with WSSV-specific primers (5′-TTGGTTTCAGCCCGAGATT-3′and 5′-CCTTGGTCAGCCCCTTGA-3′) and the WSSV-specific TaqMan probe (5′-FAM-TGCTGCCGTCTCCAA-TAMRA-3′). qRT-PCR was performed in a total volume of 25 μL, containing 12.5 μL of Premix ExTaq (TaKaRa, Japan), 0.5 μL of 10 μM forward primer, 0.5 μL of 10 μM reverse primer, 1 μL of 10 μM TaqMan fluorogenic probe, 1 μL of the DNA template, and 9.5 μL of distilled water. The predenaturation stage of the PCR program was 95 °C for 1 min, followed by the amplification stage consisting of 40 cycles of 95 °C for 30 s, 52 °C of 30 s, and 72 °C for 30 s. The assays were performed three times.

### Prediction of target genes

To predict the genes targeted by host miRNAs, the shrimp genome sequence (data unpublished) was employed with three independent computational algorithms TargetScan 5.1 (http://www.targetscan.org), miRanda (http://www.microrna.org/), and Pictar (http://pictar.mdc-berlin.de/). TargetScan was used to search for miRNA seed matches (nucleotides 2–8 from the 5′-end of miRNA) in the 3′-untranslated region (UTR) sequences. miRanda was used to match the entire miRNA sequences. The miRanda parameters were set as free energy < −20 kcal/mol and score > 50. Pictar was employed to search the combined effects of microRNA target microRNA-based or other characteristics, with score > 20. Finally, the results predicted by the three algorithms were combined, and the overlaps were calculated^33^,^49^.

### Plasmid construction

To characterize the direct interaction between miR-9041 or miR-9850 and the shrimp *STAT* gene, the 3′-UTR of *STAT* and the enhanced green fluorescent protein (EGFP) gene were cloned into a pIZ/EGFP V5-His vector (Invitrogen, USA). The EGFP gene was amplified from the pEGFP vector (BD Biosciences, USA) with EGFP-specific primers (5′-AAGAGCTCGGATCCCCGGGTA-3′and 5′-AATCTAGAGTCGCGGCCGCTTTA-3′). Subsequently, the *STAT* 3′-UTR was cloned into the pIZ vector downstream of the EGFP gene with the *Xba*I and *Sac*II restriction sites and the sequence-specific primers (5′-GCTCTAGATAATAGGTTCTAGCACATG-3′and 5′-TCCCCGCGGATGTATATTATAAAAGTTTC-3′). As controls, the *STAT* 3′-UTR sequence (GTGAATT) complementary to the miR-9041 seed sequence was mutated to TGTCCGG, thereby yielding the EGFP-∆STAT-9041 construct, whereas the *STAT* 3′-UTR sequence (TAATTTAC) complementary to the miR-9041 seed sequence was mutated to GCCGGGCA, thereby yielding the EGFP-∆STAT-9850 construct. The mutant constructs of 3′-UTRs were obtained by *Dpn*I-mediated site-directed mutagenesis (New England BioLabs, USA). All the recombinant plasmids were confirmed by sequencing.

### Cell culture, transfection, and fluorescence assays

Insect High Five cells (Invitrogen, USA) were cultured at 28 °C in Express Five serum-free medium (Invitrogen) containing l-glutamine (Invitrogen). When the cells reached 70–80% confluence, they were transfected with 6 μg of EGFP, EGFP-STAT, EGFP-∆STAT-9041, or EGFP-∆STAT-9850. Simultaneously, the cells were transfected with 300 nM of synthesized miR-9041, synthesized miR-9850, or synthesized control miRNA. All the miRNAs were synthesized by Shanghai Gene Pharma Co., Ltd. (Shanghai, China). The transfection reactions were performed in triplicate with Cellfectin transfection reagent (Invitrogen) according to the manufacturer’s protocol. After 12 h of incubation, the transfected cells were seeded into 96-well plates at a concentration of 2.0 *×* 10^4^ cells per well. At 48 h after transfection, the fluorescence of the cells was examined with a Flex Station II microplate reader (Molecular Devices, USA) at 490/510 nm for excitation and emission (Ex/Em), respectively. The fluorescence values were corrected by subtracting the autofluorescence of cells that did not express EGFP. All the experiments were biologically replicated three times.

### Synthesis of siRNAs and RNAi assay in shrimp *in vivo*

Based on the sequences of the shrimp genes *STAT*, *Dnm1*, *Dnm2*, and *Dnm3*, siRNAs were separately synthesized according to the design rule for siRNA. The siRNAs used were *STAT*-siRNA (5′-GGTAACCAGAGAACACCTT-3′), *Dnm1*-siRNA (5′-CCTTGCAGGCAAGCCTTTA-3′), *Dnm2*-siRNA (5′-GGACAATCTCCAGAGCGAA-3′), and *Dnm3*-siRNA (5′-CCATCAAGGGCGATCCCAA-3′). The sequences of siRNAs were randomly scrambled to generate the control siRNAs (*STAT*-siRNA-scrambled: 5′-AAGTCAGTTAAGCACCCGA-3′; *Dnm1*-siRNA-scrambled: 5′-TGCCCTAGTAGCCCTAAGT-3′; *Dnm2*-siRNA-scrambled: 5′-CAGATCAGCAACGGATCGA-3′; *Dnm3*-siRNA-scrambled: 5′-TCACACGGAGAAATCGCCC-3′). The formation of double-stranded RNAs was monitored by determining the size in agarose gel electrophoresis.

The RNAi assay was conducted in shrimp by the injection of a siRNA at 30 μg/shrimp into the lateral area of the fourth abdominal segment with a 1 mL sterile syringe. The siRNA (15 μg) and WSSV (10^5^ copies/mL) were co-injected into virus-free shrimp at a volume of 100 μL/shrimp. At 16 h after co-injection, the siRNA (15 μg; 100 μL/shrimp) was injected into the same shrimp. Simultaneously, the siRNAs-scrambled (15 μg; 100 μL/shrimp) was co-injected into virus-free shrimp. At 16 h after co-injection, siRNAs-scrambled (15 μg; 100 μL/shrimp) was injected into the same shrimp. WSSV (10^5^ copies/mL; 100 μL/shrimp) alone was injected as a positive control. As a negative control, PBS was injected instead of the siRNAs. For each treatment, 20 shrimp were used. Shrimp gills were collected at different times after the last injection (0, 24, 36, and 48 h). From each treatment, 5 shrimp specimens were randomly selected and collected for later use. The assays were biologically replicated three times.

### Detection of mRNA by qRT-PCR

SYBR Green fluorescent qRT-PCR was used to detect the expression of the shrimp genes *STAT*, *Dnm1*, *Dnm2*, and *Dnm3* at the mRNA level. Shrimp tissues were collected from WSSV-infected shrimp with different treatments at different time points after WSSV infection (0, 6, 12, 24, 36, and 48 h). The total RNAs were extracted from the abovementioned tissues using RNApure high-purity total RNA rapid extraction kit (Spin-column, BioTeke, Beijing, China) following the manufacturer’s protocol. RNA quality was assessed by electrophoresis on 1.0% agarose gels. The total RNA concentration was determined by obtaining the absorbance at 260 nm with a spectrophotometer. First-strand cDNA synthesis was performed with the PrimeScript® 1st Strand cDNA Synthesis Kit (Takara, Dalian, China) and the Oligo dT Primer. The cDNA mix was diluted to 1:10 by PCR-grade water before storage at −80 °C until qRT-PCR. The shrimp *glyceraldehyde 3-phosphate dehydrogenase* (*GAPDH*) gene was used as a control. The gene-specific primers (*STAT*-RT-F: 5′-GTGTGAACGACATCAGACCAAG-3′, *STAT*-RT-R: 5′-TAACAAGTGAAGAGAGAAGACGAGT-3′; *Dnm1*-RT-F: 5′-TTTTACACAAGAAGGGCGACAGG-3′, *Dnm1*-RT-R: 5′-CACATTTGGCGAATAAACCCG-3′; *Dnm2*-RT-F: 5′-CACAACACCTACGCATCAGCTTC-3′, *Dnm2*-RT-R: 5′-CACAGTCCTTGGTCTCCCTCTCC-3′; *Dnm3*-RT-F: 5′-GGTCCATATCACATTGCATCAT-3′, *Dnm3*-RT-R: 5′-TTCTCAACTCAACCTCCTTTCTC-3′; *GAPDH*-RT-F: 5′-TGCCGCCCAGAACATCATT-3′, *GAPDH*-RT-R: 5′-TCGTCTTCGGTGTAGCCCA-3′) were used. qRT-PCR was performed according to the manufacturer’s instructions for the 2 × SYBR Premix Ex Taq Kit (Takara, Japan) with a real-time thermal cycler (Bio-Rad, Hercules, CA, USA). PCR was conducted with initial denaturation at 95 °C for 3 min, followed by 40 cycles at 95 °C for 15 s and at 60 °C for 30 s. Data were quantified by the 2^−ΔΔCT^ (ΔΔCT = ΔCT of sample − ΔCT of untreated control) method^50^ and subjected to statistical analysis.

### Recombinant expression and purification of Dnm1, Dnm2, and Dnm3 in *Escherichia coli*

The full-length cDNAs encoding the mature Dnm1, Dnm2, and Dnm3 were amplified by the primers (Dnm1-ex-F: 5′-TATCGGATCCGAATTCCTGCAAGATGCATTTACACAG-3′, Dnm1-ex-R: 5′-GGTGGTGGTGCTCGAGATGTGGGCGCTGAGGCACTTGA-3′; Dnm2-ex-F: 5′-TATCGGATCCGAATTCCAGGATGTCTTCAACACCGTC-3′, Dnm2-ex-R: 5′-GGTGGTGGTGCTCGAGGATTTCACCAATAATCACGTTAGC-3′; and Dnm3-ex-F: 5′-TATCGGATCCGAATTCGGAGTTAGCCAGTCACGGTG-3′, Dnm3-ex-R: 5′-GGTGGTGGTGCTCGAGTGACAGAGCATCTTCCCACTG-3′), with *EcoR*I and *XhoI* sites in the forward and reverse primers, respectively. The amplified products were cloned into pET30a(+) vector (Novagen) and transformed into competent cells of *E. coli* BL21 (DE3) (TransGen Biotech, Beijing, China). The positive clones were screened by PCR with specific primers and confirmed by further nucleotide sequencing. The cells were grown at 37 °C in LB medium under agitation until they reached an OD_600 nm_ of 0.6–0.8. Recombinant expression was induced by the addition of isopropyl-b-d-1-thiogalactopyranoside (IPTG; 0.5 mmol/L), followed by incubation for another 4 h under the same conditions. The recombinant rDnm1, rDnm2, and rDnm3 proteins were separated by reducing 12.5% SDS polyacrylamide gel electrophoresis (SDS–PAGE) and visualized with Coomassie brilliant blue R250. rDnm1, rDnm2, and rDnm3 with a His tag were purified by His Bind resin chromatography (Novagen, USA) according to the manufacturer’s instructions.

### Inhibition of WSSV copies

A total of 500 μL purified recombinant rDnm1 (600 μg/mL), rDnm2 (600 μg/mL), or rDnm3 (600 μg/mL) was individually incubated with 500 μL of WSSV (10^5^ copies/mL) at 28 °C for 30 min with rotation. Bovine serum albumin (BSA; 600 μg/mL, 500 μL) was incubated with WSSV as the control. Four groups of mixtures (100 μL each) were respectively injected into the shrimp. The gills of 5 shrimp were collected at different post-injection times (0, 24, and 48 h) to detect the WSSV copies by qRT-PCR.

### Statistical analysis

The data from three independent experiments were analyzed by one-way ANOVA to calculate the mean and standard deviation of the triplicate assays. Statistically significant differences between treatments were determined with the *t*-test, with significance defined as *P* < 0.05 or *P* < 0.01.

## Additional Information

**How to cite this article**: Huang, Y. *et al*. Two host microRNAs influence WSSV replication via *STAT* gene regulation. *Sci. Rep*. **6**, 23643; doi: 10.1038/srep23643 (2016).

## Supplementary Material

Supplementary Information
