# Supplementary material for: Two host microRNAs influence WSSV replication via STAT gene regulation
Source: Sci Rep. 2016 Mar 31;6:23643. doi: 10.1038/srep23643 (PMC4814834; doi:10.1038/srep23643)
Supplement: Supplementary Information [file srep23643-s1.doc]

**Title page**

**Two host microRNAs influence WSSV replication via *STAT* gene regulation**

Ying Huang^1^, Wen Wang^1^, Qian Ren^1^*

1. Jiangsu Key Laboratory for Biodiversity & Biotechnology and Jiangsu Key Laboratory for Aquatic Crustacean Diseases, College of Life Sciences, Nanjing Normal University, Nanjing 210046, China

* Corresponding author: Dr. Qian Ren

Tel: 86-25-85891955

E-mail: [renqian0402@126.com](mailto:renqian0402@126.com)

Fig S1


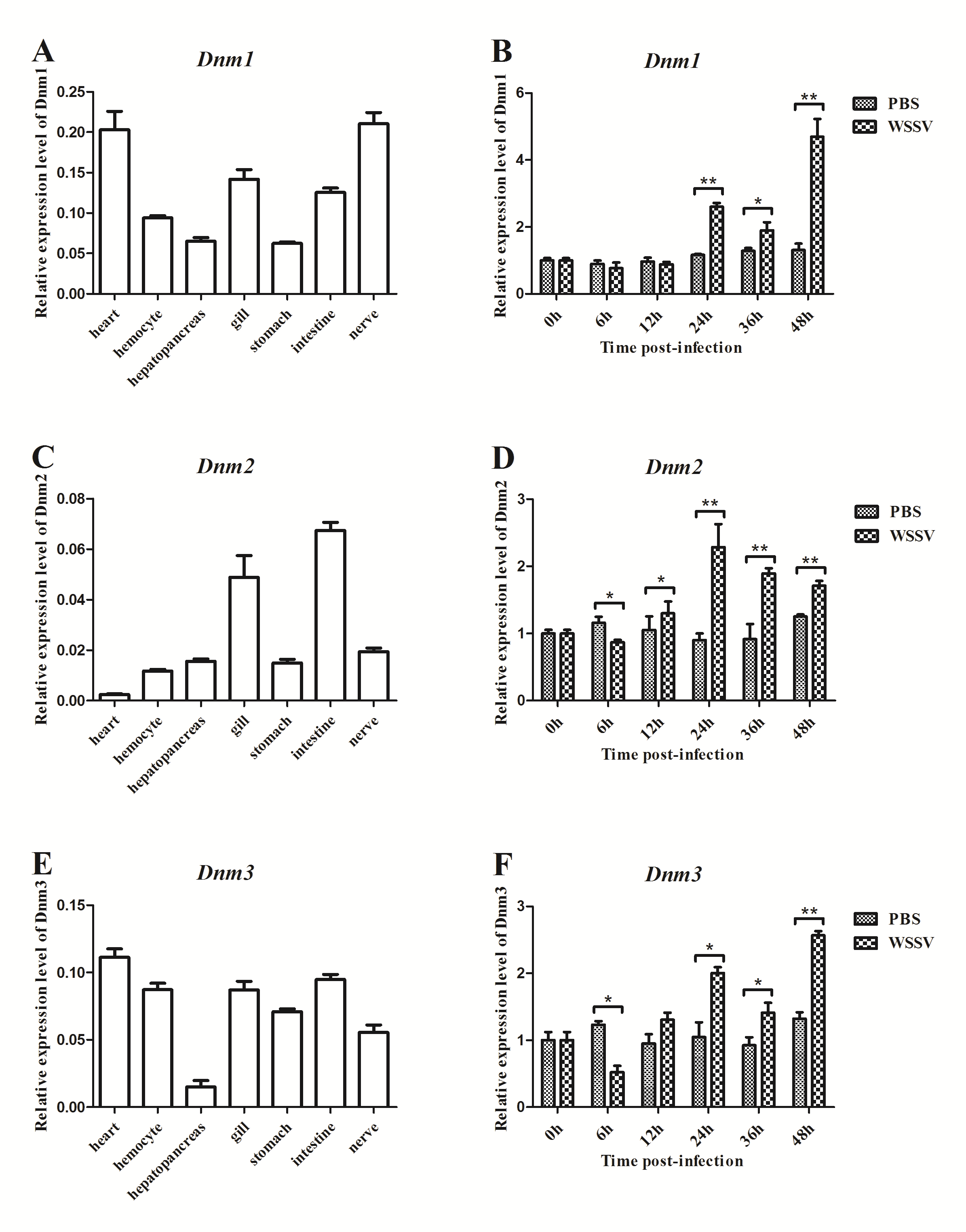


**Fig S1.** **The roles of *Dnm1* to *Dnm3* in virus infection.** The distributions of *Dnm1* (A), *Dnm2* (C) and *Dnm3* (E) in various tissues of shrimp. The shrimp *Dnms* mRNA levels of heart, hemocyte, hepatopancreas, gill, stomach, intestine and nerve were examined using quantitative real-time PCR. Shrimp GAPDH was used as a control to calibrate the cDNA template for all the samples. The expression profile of *Dnm1* (B), *Dnm2* (D) and *Dnm3* (F) in shrimp in response to virus infection. Shrimp were infected with WSSV. At different time post-infection (0, 6, 12, 24, 36, and 48 h), the *Dnms* expressions in gills was detected with quantitative real-time PCR.
